# A Quantitative Comparison of Clinically Employed Parameters in the Assessment of Acute Cerebral Ischemia Using Dynamic Susceptibility Contrast Magnetic Resonance Imaging

**DOI:** 10.3389/fphys.2018.01945

**Published:** 2019-01-15

**Authors:** Christian Nasel, Uros Klickovic, Heike-Marie Kührer, Kersten Villringer, Jochen B. Fiebach, Arno Villringer, Ewald Moser

**Affiliations:** ^1^Center for Medical Physics and Biomedical Engineering, Medical University of Vienna, Vienna, Austria; ^2^Department of Radiology, University Hospital Tulln, Tulln, Austria; ^3^MR Center of Excellence, Medical University of Vienna, Vienna, Austria; ^4^Sobell Department of Motor Neuroscience and Movement Disorders, UCL Queen Square Institute of Neurology, University College London, London, United Kingdom; ^5^Center for Stroke Research Berlin, Neuroradiology, Charité-Universitätsmedizin, Berlin, Germany; ^6^Department of Cognitive Neurology, University Hospital Leipzig, Leipzig, Germany; ^7^Department of Neurology, Max Planck Institute for Human Cognitive and Brain Sciences, Leipzig, Germany

**Keywords:** cerebral ischemia, cerebral circulation, perfusion magnetic resonance imaging, contrast media, ischemic stroke

## Abstract

**Purpose:** Perfusion magnetic resonance imaging (P-MRI) is part of the mismatch concept employed for therapy decisions in acute ischemic stroke. Using dynamic susceptibility contrast (DSC) MRI the time-to-maximum (Tmax) parameter is quite popular, but its inconsistently defined computation, arterial input function (AIF) selection, and the applied deconvolution method may introduce bias into the assessment. Alternatively, parameter free methods, namely, standardized time-to-peak (stdTTP), z_f_-score, and standardized-z_f_ (stdZ) are also available, offering consistent calculation procedures without the need of an AIF or deconvolution.

**Methods:** Tmax was compared to stdTTP, z_f_-, and stdZ to evaluate robustness of infarct volume estimation in 66 patients, using data from two different sites and MR systems (i.e., 1.5T vs. 3T; short TR (= 689 ms) vs. medium TR (= 1,390 ms); bolus dose 0.1 or 0.2 ml/kgBW, respectively).

**Results:** Quality factors (QF) for Tmax were 0.54 ± 0.18 (sensitivity), 0.90 ± 0.06 (specificity), and 0.87 ± 0.05 (accuracy). Though not significantly different, best specificity (0.93 ± 0.05) and accuracy (0.90 ± 0.04) were found for stdTTP with a sensitivity of 0.56 ± 0.17. Other tested parameters performed not significantly worse than Tmax and stdTTP, but absolute values of QFs were slightly lower, except for z_f_ showing the highest sensitivity (0.72 ± 0.16). Accordingly, in ROC-analysis testing the parameter performance to predict the final infarct volume, stdTTP and z_f_ showed the best performance. The odds for stdTTP to obtain the best prediction of the final infarct size, was 6.42 times higher compared to all other parameters (odds-ratio test; *p* = 2.2^*^10–16).

**Conclusion:** Based on our results, we suggest to reanalyze data from large cohort studies using the parameters presented here, particularly stdTTP and zf-score, to further increase consistency of perfusion assessment in acute ischemic stroke.

## Introduction

Dynamic susceptibility contrast magnetic resonance imaging (DSC-MRI) provides good contrast at short imaging times, but lacks commonly accepted robust methods to quantitatively assess cerebral perfusion. For instance, calculation of quantitative cerebral blood flow (CBF), mean transit time (MTT), but also the nowadays often used surrogate parameter time-to-maximum (Tmax), require deconvolution of a voxel's time-concentration curve (TC) with a manually or automatically selected arterial input function (AIF) (Carroll et al., 2002; Ostergaard, 2005; Boutelier et al., 2012). Selection of an AIF as well as deconvolution are prone to methodological bias potentially leading to significant variation of the results even when evaluating the same DSC-data (Zaro-Weber et al., 2009, 2012). Moreover, optionally introduced TC-model fitting prior to deconvolution was also found to alter the appearance of the finally depicted lesion on the resulting perfusion map (Christensen et al., 2009; Forkert et al., 2013).

Parameters derived from direct assessment of the TC, like absolute time-to-peak (TTP) or relative TTP (relTTP), were initially considered less meaningful, but were recently shown to potentially perform even better than AIF-based techniques (Yamada, 2002; Christensen et al., 2009; Zaro-Weber et al., 2009). However, unequivocal results are still not warranted by this direct assessment, since accepted calculation modifications, like optional TC-model fitting, also significantly alter the finally depicted lesion size (Forkert et al., 2013). Additionally, most peak enhancement time related parameters require time thresholds to differentiate regular from critical perfusion, where for Tmax various thresholds, ranging at least from 4 to 8 s in the human brain, are currently under discussion (Olivot et al., 2009; Forkert et al., 2013).

Another approach in cerebral perfusion assessment is to analyze the spatiotemporal distribution of absolute TTP without manipulating the TC in order stay as close as possible to physiology (Nasel et al., 2000, 2014). Parameters based on this approach require neither TC-curve fitting nor an AIF-based deconvolution. This, additionally, eliminates major sources of methodically introduced bias and yields only one distinct and robust result when calculating the perfusion map. Two distribution-parameters evaluating the respective absolute TTP-histogram have been introduced: (a) standardized TTP (stdTTP), estimating the temporal absolute TTP-relations in separate volumes of interest (VOI), and (b) a generic *z*_*f*_-score describing the global absolute TTP distribution over time in the whole brain (Nasel et al., 2000, 2001). A combination of both approaches, called standardized *z*_*f*_ (*stdZ*), is also presented in this study. While the unit of stdTTP still is second, i.e., time, the *z*_*f*_-score and *stdZ* are of pure statistical nature and, therefore, dimensionless (Nasel et al., 2004, 2014, 2017). StdTTP, *z*_*f*_-score, and *stdZ*, all require thresholds for the differentiation of critical perfusion, however, only one distinct threshold was defined for each parameter.

In this study, data from two different clinical units running different MR-scanners and protocols was used. We assessed the performance of the distribution parameters stdTTP, *z*_*f*_ and *stdZ* as compared to the AIF-based Tmax parameter that proved useful in large clinical trials investigating acute cerebral ischemia (Lansberg et al., 2012). Therefore, we evaluated and compared the ability of stdTTP, *z*_*f*_, *stdZ*, and Tmax to correctly indicate the final infarct volume and assessed the chance for each parameter to provide the best final infarct prediction.

## Methods

### Patients

In total 66 multi-parametric MRI examinations from two experienced clinical centers (group 1: *n* = 32; group 2: *n* = 34) were included in the study. In both centers consecutive patients suffering acute ischemic thrombo-embolic stroke with occlusions of the M1–M4 segments of one middle cerebral artery were collected in a prospective fashion. Stroke treatment was performed either as intravenous thrombolytic therapy with 0.9 mg/kg_BW(bodyweight)_ of recombinant tissue plasmin activator (rTPA) or as endovascular therapy with thrombectomy and/or thrombus aspiration. Combinations of both were also possible. Since therapeutical effects and stroke characteristics equally affected calculation of all tested perfusion parameters in the study no further distinction concerning this aspect was made. A full summary of the patients' characteristics is provided in Table 1.

**Table 1:** Demographic and treatment data of patients included into the study.

	**Group 1 (*n* = 32)**	**Group 2 (*n* = 34)**
DSC-protocol	3 T: TR = 1,390 ms TE = 29 ms	1.5 T: TR = 689 ms TE = 17 ms
F/m	10/22	13/21
Age [years]	72 ± 11	66 ± 13
Smoking	16%	35%
Atrial fibrillation	25%	26%
Hyperlipidemia	28%	50%
Hypertension	72%	71%
Diabetes	22%	29%
iv. thrombolysis	50%	62%
Thrombectomy	0.03%	79%
Time to treatment [h]	3.00 ± 2.82 (IQR: 8.45)	3.00 ± 1.48 (IQR: 2.35)
Final infarct volume [cm^3^]	35.45 ± 40.48 (IQR: 85.14)	58.56 ± 63.42 (IQR: 130.33)
Final TICI score (in group 2 only patients receiving thrombectomy were rated)	NA … 3% 0 … 6% 2a … 22% 2b … 13% 3 … 56%	NA … 35% 0 … 3% 2a … 3% 2b … 12% 3 … 61%
Modified Rankin scale after (day 90)	0 … 16% 1 … 22% 2 … 16% 3 … 16% 4 … 28% 5 … 6% 6 … 13%	0 … 12% 1 … 24% 2 … 9% 3 … 15% 4 … 18% 5 … 12% 6 … 12%

*Descriptive statistics are given as median ± MAD until not indicated otherwise*.

As far as applicable, written informed consent was obtained from all patients. The study was approved by the Lower Austrian Ethics Commission (GS1-EK-4/512-2017) and was performed according to rules and regulations of the World Medical Association-council recommendations of ethical principles for medical research involving human subjects (World Medical Association, 2014).

### Magnetic Resonance Imaging

All patients received multi-parametric MRI, including DSC-MRI, during the acute phase of ischemic stroke, and received a control examination between days 1 and 30 after the acute event, either using multi-parametric MRI (group 1: *n* = 32; group 2: *n* = 27) or computed tomography (group 2: *n* = 7).

DSC-MRI in group 1 (i.e., patients from center 1) was performed as dynamic contrast enhanced T2^*^-weighted single shot, gradient echo (GE), echo planar imaging (EPI) sequence on a clinical 3 T MR-scanner (TIM TRIO, Siemens Medical Systems, Erlangen, Germany) with 29 ms/1,390 ms/60° (TE/TR/flip angle). This enabled acquisition of 80 stacks consisting of 21 slices (0.5 mm gap) resulting in a nominal voxel size of 1.8 × 1.8 × 5 mm, within a total of 118 s. All examinations in group 2 (i.e., patients from center 2) were performed on a clinical 1.5 T MR-scanner (Avanto, Siemens Medical Systems, Erlangen, Germany), where DSC-MRI was performed using a time optimized, short-TR, single shot T2^*^w-GRE-EPI-sequence with 17 ms/689 ms/35° (TE/TR/flip angle), allowing the acquisition of 81 stacks consisting of 20 slices (0.6 mm gap) with a reconstructed voxel size of 1.15 × 1.15 × 6 mm (acquired voxel size: 2.3 × 2.5 × 6 mm), within a total of 60 s. Gd-based contrast agents (center 1: Gadobutrol 1.0 mmol/ml, dosage: 0.1 ml/kg_BW_, Bayer® Austria; center 2: gadoterate meglumine 0.5 mmol/ml, dosage: 0.2 ml/kg_BW_, Guerbet® Austria), injected by an automatic injector at a delay of 10 s into a cubital vein at a flow rate of 5 ml/s, followed by a flush of 20 ml saline, were administered. The sampling rates of the global brain bolus passage were, therefore, 0.72 Hz (TR = 1,390 ms), and 1.45 Hz (TR = 689 ms), respectively.

Furthermore, both multi-parametric imaging protocols included diffusion weighted imaging (dual-b SE-EPI: b1 = 0 s/mm^2^; b2 = 1,000 s/mm^2^), MR-angiography and conventional MRI performed either as enhanced PD/T2w-IR- or flair imaging (Nasel, 2005). In follow-up examinations either the initial MRI protocol was repeated or computed tomography (i.e., isotropic spiral scan with a voxel size of 0.5 mm^3^) was performed.

### Calculation of Time to Maximum

According to the respective software documentation for the Tmax estimation, fitting of the TC-curve of each voxel in order to better determine the bolus arrival time and to eliminate TR related discretization errors was performed (Calamante et al., 2010). Subsequently, TC-curves acquired at TR = 1,390 ms were deconvoluted, after automatic AIF-selection, using standard singular value decomposition (sSVD) (Ostergaard et al., 1996), while oscillation index regularized block-circulant SVD (oSVD) was used for those recorded at TR = 689 ms. Other than sSVD, the oSVD-variant is considered as delay insensitive, and more details about both methods can be found elsewhere (Ostergaard et al., 1996; Wu et al., 2003). Tmax-maps were calculated using commercially available software packages (center 1: StrokeTool®, Digital Image Solutions, Dr. Hans-Jörg Wittsack, Germany; center 2: olea sphere 2.4®, olea medical, France).

### Calculation of Standardized Time to Peak

For the calculation of stdTTP, z_*f*_, and *stdZ*, at first plain mean curve smoothing was applied to all TCs and, thereafter, voxel-wise calculation of absolute TTP-values was performed. The envelope of the main peak of the global TTP-histogram describing the first pass of the administered contrast agent represents the so-called TTP distribution curve (TDC), which provides meaningful information about the bolus distribution over time in the brain (Nasel et al., 2014). While the TDC reflects predominantly the temporal relations of absolute TTP-values, spatial relations between absolute TTP-values of individual voxels are mostly neglected. Spatial relations of absolute TTP-values within the same slice are most relevant, however, delays caused by varying contrast arrival times in different slices may be not. Using a simple and robust spatial standardization step renders absolute TTP quite insensitive to spurious delays (Nasel et al., 2000). This is achieved by relating absolute TTP of spatially correlated voxels, arranged in a certain ***VOI***, to a ***VOI***-specific arrival time offset ${oTTP}_{VOI}^{-}$ that is calculated as:

(1) $$oTTP_{VOI}^{-}=Q_{VOI}\left(0.03\right)\text{ with }Q_{VOI}=TDC_{V(x,y,z\in VOI)}$$

In Equation 1 ***VOI*** usually corresponds to the respective slice measured and ***Q***_***VOI***_ is the associated quantile function described by the VOI-specific ***TDC***_(x, y, z∈***VOI***)_, which is the density function of absolute TTP of all voxels **V**(x, y, z) contained in ***VOI***. By definition ${oTTP}_{VOI}^{-}$ is calculated as the lower 3%-quantile of **TDC**_**V**(x, y, z∈**VOI**)_. Finally, the subtraction of ${oTTP}_{VOI}^{-}$ from every absolute voxel-TTP in ***VOI*** provides stdTTP:

(2) $$\begin{array}{l} stdTTP_{\text{V}(\text{x},\text{y},\text{z}\in VOI\text{)}}:\;=\\ \{\begin{array}{c} ttp_{\text{V}(\text{x},\text{y},\text{z}\in VOI\text{)}}-oTTP_{VOI}^{-}\;for\;ttp_{\text{V}(\text{x},\text{y},\text{z}\in VOI\text{)}}\geq oTTP_{VOI}^{-}\\ \;0\;for\;ttp_{\text{V}(\text{x},\text{y},\text{z}\in VOI\text{)}}<oTTP_{V\text{O}I}^{-} \end{array} \end{array}$$

In Equation 2 ***ttp*_V(x, y, z_ ∈ ***VOI***)** denotes the absolute TTP of all voxels ***V***(x, y, z) of ***VOI*** and ***stdTTP*_V(x, y, z∈***VOI***)_** is the corresponding, standardized TTP-value. All other symbols have the same meaning as in Equation 1.

**Figure 1:**
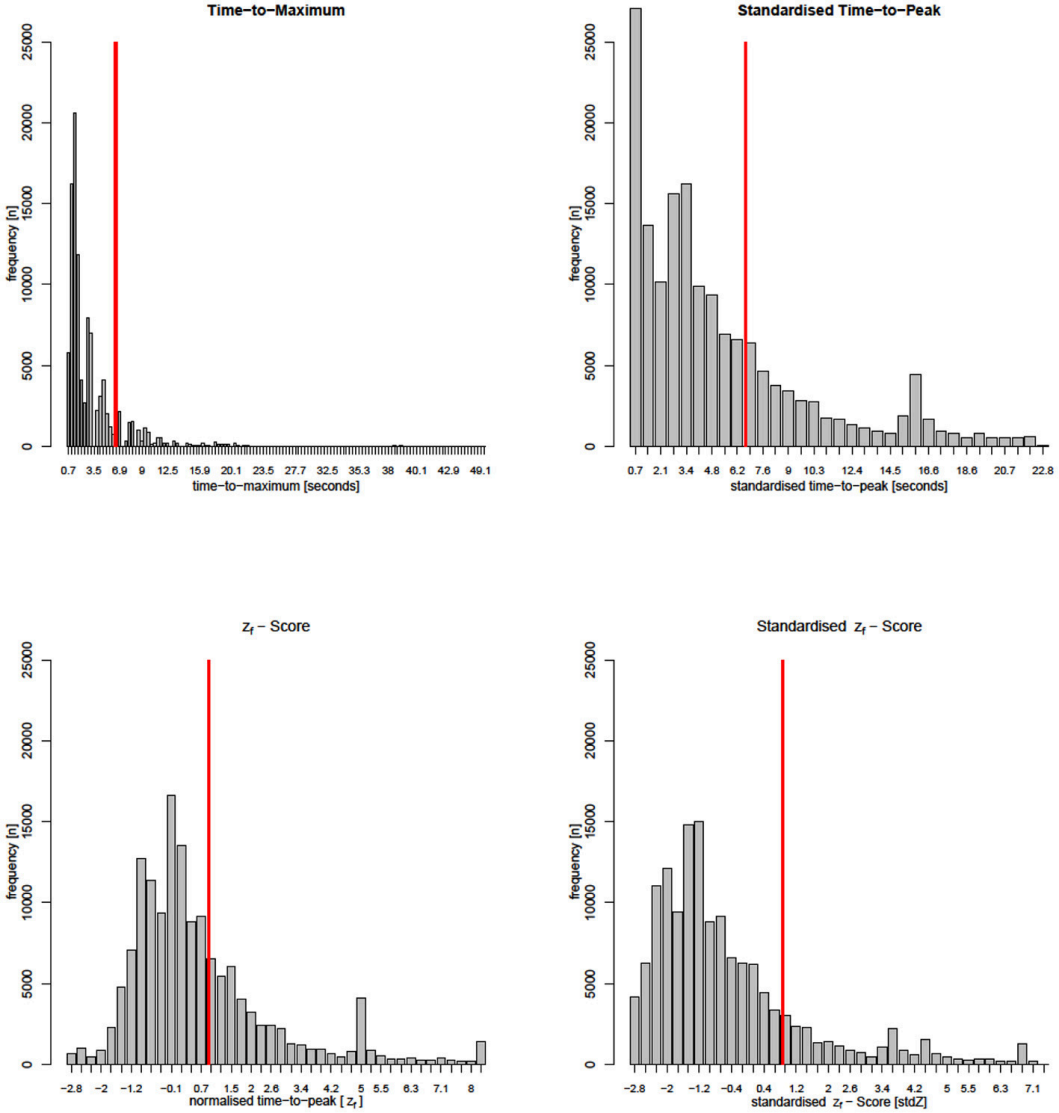
Parameter-histograms from a single patient of group 2 (critical perfusion thresholds are marked by red lines). Note that the Tmax-calculation interpolates many time steps not directly measured, while all other parameters stay close to the DSC-MRI measurement. Distributions of Tmax and stdTTP appear very similar. The std*Z*_*f*_ -distribution still shares many similarities with Tmax and stdTTP histograms, but the distribution of *z*_*f*_ that resembles the actual TTP-histogram is quite different.

Technically, this standardization step simulates the simultaneous filling of all ***VOIs***. This enables the direct comparisons of stdTTP-values between different examinations as spurious run time delays are largely eliminated (Nasel et al., 2000, 2004). Note that by this standardization not only absolute TTP is transformed to stdTTP, but also a *new* spatiotemporal stdTTP-distribution is generated (Figure 1). StdTTP and TDC raw data files were calculated using in-house developed software (jPerfusionModule, v 3.1; available upon request from the corresponding author, RRID:SCR_016534).

### Calculation of *z*_*f*_-Scores

In terms of absolute TTP-values the global TDC of different examinations is not directly comparable. Normalization through fitting of a double Gaussian model to a given global TDC was recently shown to solve this problem, where the individual global TDC is replaced by a generic density function ***TDC***_***f***_ (Nasel et al., 2014):

(3) $$TDC_{f}=\sum_{i=1}^{2}k_{i}\int_{-\infty }^{TTP}\frac{1}{\sigma_{i}\sqrt{2\pi }}e^{-\frac{{\left(ttp-\mu_{i}\right)}^{2}}{2\sigma_{i}^{2}}}dttp\;with\;1=\sum_{i=1}^{2}k_{i}.$$

Parameters **μ_i_**, **σ_i_**, and ***k***_*i*_ in Equation 3 denote the mean, the standard deviation and the probability weight of the respective Gaussian sub-function of the applied double Gaussian model. Quantitative estimates of **μ**_**i**_ and **σ**_**i**_ are obtained via finite mixture modeling with incorporation of the expectation-maximization algorithm (Gruen and Leisch, 2008). *TDC*_*f*_ transforms the absolute TTP from a correlated global TDC to distinct generic model-based z-quantiles, referred to as **z**_**f**_-score:

(4) $${\text{z}}_{f}:=\sum_{i=1}^{2}k_{i}\frac{ttp-\mu_{i}}{\sigma_{i}}\;\text{with}p(z_{f})=TDC_{f}(z_{f}).$$

Parameters ***ttp***, **μ_i_**, **σ**_i_, and ***k_i_*** in Equation 4 have the same meaning as in equation 3. ***TDC_f_*** represents the density function ***p***(z_*f*_) of the correlated global TDC, providing generic z_*f*_-quantiles (scores) that can be used to directly compare measurements of different examinations (for more details see Nasel et al., 2014, 2017). Note that in contrast to standardization, normalization preserves the original distribution of absolute TTP-values (Figure 1).

### Calculation of Standardized **z*_*f*_*-Scores

Since ***TDC***_***f***_ simply models the TDC, the derived *z*_*f*_-scores are not a priori corrected for spurious delays inherent to the measurement. Introducing z_*f*_ instead of ***ttp*** into equations 1 and 2 standardizes z_*f*_ and leads to *stdZ*.

(5) $$stdZ:=\{\begin{array}{c} {\text{z}}_{f_{V(x,y,z\in VOI)}}-{\mathrm{oz}}_{f_{VOI}}{}^{-}for\;{\text{z}}_{f_{V(x,y,z\in VOI)}}\geq {\mathrm{oz}}_{f_{VOI}}{}^{-}\\ \;0\;for\;z_{f_{V(x,y,z\in VOI)}}<{\mathrm{oz}}_{f_{VOI}}{}^{-} \end{array}$$

In Equation 5 z_*f*_*V*(**x, y, z**∈**VOI**)__ denotes the *z*_*f*_-scores of all voxels V(x, y, z) ∈ *****VOI*****, which are related to the ***VOI***-specific quantile offset ${{\mathrm{oz}}_{f_{VOI}}}^{-}$ that is otherwise calculated in the same way as the stdTTP-offset described in equation 1, except for replacing ***TDC***_**V**(x, y, z∈***VOI***)_ of the original stdTTP calculation by the ***VOI***-specific corresponding fitted ***TDC***_***f***_V(x, y, z∈**VOI**)__. Accordingly, ${{\mathrm{oz}}_{f_{VOI}}}^{-}$ is calculated as the lower 2.275%-quantile of the newly introduced ***TDC***_***f***_V(x, y, z∈**VOI**)__, which includes all measured voxels with z_*f*_-scores in the interval [−3,+∞] into the analysis.

### Assessment of Ischemia

Recent ischemic infarcts were identified by an experienced neuroradiologist (>20 years; C.N.) in follow-up MRI or CT scans as T2w-high signal or hypodense areas, respectively, correlated to the initial diffusion restriction shown in acute DWI. If available, follow-up DWI was additionally considered. Regions of interest (ROIs) covering the infarcted volume were carefully drawn in follow-up examinations and superposed by rigid body transformations on the perfusion maps (Tmax, *z*_*f*_-score, and *stdZ*) of the acute phase. Another ROI covering all parts of the supratentorial regular brain was drawn on spatially averaged raw data images directly derived from T2^*^w-EPI perfusion MRI acquired during the acute phase, which were already in alignment with the perfusion maps. This way, assessment of parameter performance in ischemic and regular regions in the vascular territory affected by the acute infarct and the comparable contralateral side was feasible. Non-recent lesions were excluded. Thresholds for critical perfusion were for Tmax > = 6 s, for stdTTP > = 7 s, and for z_*f*_ as well as *stdZ* > = IPv (Nasel et al., 2004, 2014; Zaro-Weber et al., 2010). Here, IPv denotes the venous inflection point of the respective model fit *****TDC***_***f***_** and was chosen because it marks the time point where the katacrotic part of the originally measured TDC changes its curvature during wash out depending on **μ**_**i**_, **σ**_**i**_, and **k**_**i**_ as described in Equation 3. ROIs were created using freely available image viewing software (MRIcro V1.40, RRID: SCR_008264) (Rorden and Brett, 2000). Rigid body transformations were performed using the software package SPM12 (Statistical Parameter Mapping 12, UCL-Wellcome Trust center for Neuroimaging, London, UK, RRID: SCR_007037).

### Statistical Analysis

Descriptive statistics are given as median and MAD (mean absolute deviation), if not mentioned otherwise. Data quality of Tmax, stdTTP, z_*f*_, and *stdZ* was considered rationally scaled. In inferential statistical analysis differences of 0.05 were considered significant, and corrections for multiple comparisons were performed in the *post-hoc* analysis if applicable (method: Bonferroni).

Sensitivity, specificity and accuracy were assessed as quality factors for all perfusion parameters on a voxel-by-voxel basis and their behavior was evaluated using non-parametric tests considering non-normality and heteroscedasticity of the tested variables in the various groups after Shapiro-Wilk and Bartlett testing. Based on accuracy, which considers both, precision and trueness, we also calculated the odds for each perfusion parameter to give the best infarct prediction. Due to the small sample size no subgroup analyses were performed in order to allow further statistical testing for significant differences. Thus, statistical outcome is independent of the protocol employed. Odds-ratios and 95%-intervals of confidence (CI_95%_) of the whole sample are given as logarithmic quantities. The chance to get the best result by each parameter was tested against a ground truth derived from the cumulative accuracies of all other parameters using odds-ratio tests (Meyer et al., 2017). Receiver-operator characteristics (ROC) of parameter performance depending on the individual infarct characteristics were assessed within groups and cumulative by calculating the area under the curve (AUC) and CI_95%_, thereby incorporating testing according to DeLong et al. (1988) (CI_95%_).

For statistical assessment and calculation of z_*f*_- and*stdZ* the software package R (version 3.2.0, RRID: SCR_003005) was used with in-house developed R-scripts employing packages: “flexmix,” “pROC,” “FSA,” “DTK,” “vcd,” and “AnalyzeFMRI” (Gruen and Leisch, 2008; Bordier et al., 2011; Robin et al., 2011; Lau, 2013; R Development CoreTeam, 2015; Meyer et al., 2017; Ogle, 2018).

## Results

**Figure 2:**
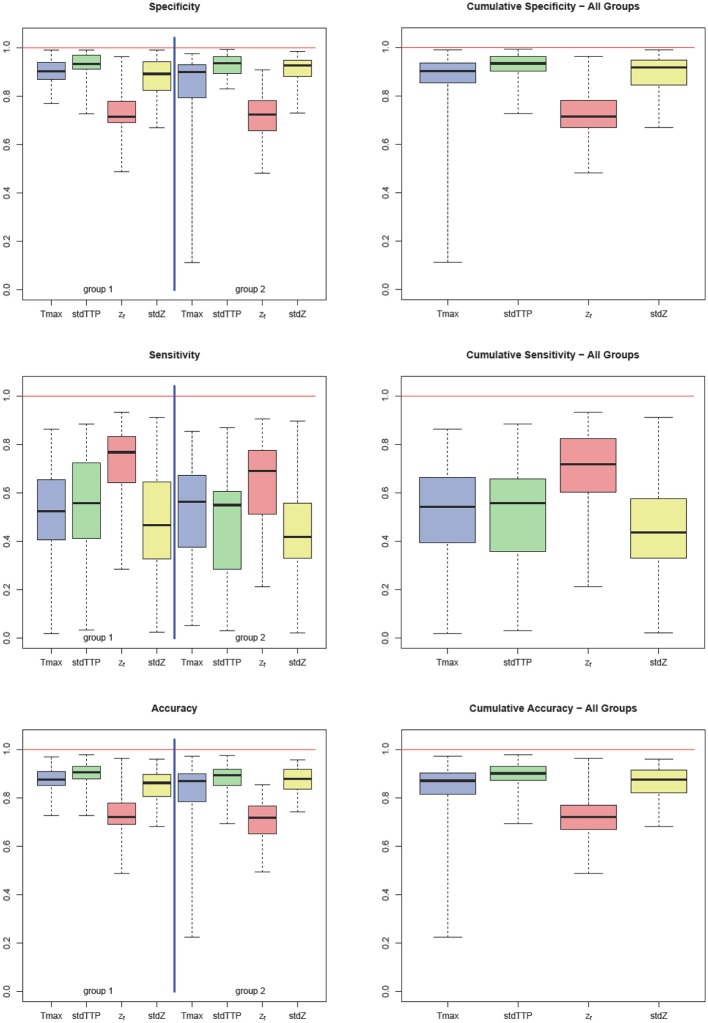
Quality factors: sensitivity, specificity, and accuracy for Tmax (blue), stdTTP (green), *z*_*f*_ (red), and stdZ (yellow) by group (left part) and cumulative (right part). Note that stdTTP shows the highest specificity and the best overall performance, whereas *z*_*f*_ shows the highest sensitivity.

Sensitivity in group 1 was 0.52 ± 0.19 for Tmax, 0.56 ± 0.23 for stdTTP, 0.77 ± 0.11 for z_*f*_ and 0.47 ± 0.22 for *stdZ*, respectively. Specificity obtained for group 1 was 0.90 ± 0.05 for Tmax, 0.93 ± 0.04 for stdTTP, 0.71 ± 0.07 for z_*f*_ and 0.89 ± 0.08 for *stdZ*, respectively. For group 2, sensitivity was 0.56 ± 0.19 for Tmax, 0.55 ± 0.17 for stdTTP, 0.69 ± 0.16 for z_*f*_ and 0.42 ± 0.20 for *stdZ*, while specificity was 0.90 ± 0.06 for Tmax, 0.94 ± 0.05 for stdTTP, 0.72 ± 0.09 for z_*f*_ and 0.93 ± 0.06 for *stdZ*, respectively. Cumulative sensitivity obtained was 0.54 ± 0.18 for Tmax, 0.56 ± 0.17 for stdTTP, 0.72 ± 0.16 for z_*f*_ and 0.44 ± 0.18 for *stdZ* with a specificity of 0.90 ± 0.06 for Tmax, 0.93 ± 0.05 for stdTTP, 0.71 ± 0.08 for z_*f*_ and 0.92 ± 0.06 for *stdZ*, respectively. Accuracy for group 1 was 0.87 ± 0.04 for Tmax, 0.91 ± 0.04 for stdTTP, 0.72 ± 0.06 for z_*f*_ and 0.86 ± 0.07 for *stdZ*, respectively. For group 2 an accuracy of 0.87 ± 0.07 for Tmax, 0.89 ± 0.05 for stdTTP, 0.72 ± 0.08 for z_*f*_ and 0.88 ± 0.06 for *stdZ* was obtained. Finally, cumulative accuracy was 0.87 ± 0.05 for Tmax, 0.90 ± 0.04 for stdTTP, 0.72 ± 0.07 for z_*f*_ and 0.87 ± 0.06 for *stdZ*, respectively (Figure 2).

Analyzing the differences between quality factors related to the various perfusion parameters in both groups revealed an exceptional behavior of the z_*f*_-score. In group 1 z_*f*_ showed a significantly lower specificity compared to all other parameters, while its sensitivity was significantly higher than that of Tmax and *stdZ* [Kruskal-Wallis test; *post-hoc—*analysis: DTK-test; *n* = 32; specificity (z_*f*_ < Tmax, stdTTP,*stdZ*): *p* < 0.001 sig. [corr.]; sensitivity (z_*f*_>Tmax,*stdZ*): *p* < 0.001 sig. [corr.]]. In group 2, specificity of z_*f*_ was significantly lower than that of stdTTP and *stdZ*, and sensitivity of z_*f*_ was significantly higher than that of *stdZ* [Kruskal-Wallis test; *post-hoc*—analysis: DTK-test; *n* = 34; specificity (z_*f*_ < stdTTP,*stdZ*): *p* < 0.001 sig. [corr.]; sensitivity (z_*f*_>*stdZ*): *p* < 0.001 sig. [corr.]]. Cumulative analysis of quality factors of the perfusion parameter z_*f*_ showed a similar behavior with a significantly lower specificity as compared to stdTTP and *stdZ*, while sensitivity was significantly higher compared to all other perfusion parameters [Kruskal-Wallis test; *post-hoc—*analysis: DTK-test; *n* = 66; cumulated specificity (z_*f*_ < stdTTP, *stdZ*): *p* < 0.001 sig. [corr.]; cumulated sensitivity (z_*f*_>Tmax, stdTTP, *stdZ*): *p* < 0.001 sig. [corr.]]. Accuracy of z_*f*_ was significantly lower in group 1 compared to Tmax, stdTTP, and *stdZ* [Kruskal-Wallis test; *post-hoc—*analysis: DTK-test; *n* = 32; accuracy (z_*f*_ < Tmax, stdTTP,*stdZ*): *p* < 0.001 sig. [corr.]], while in group 2 the accuracy of z_*f*_ was significantly lower compared to stdTTP and *stdZ* only [Kruskal-Wallis test; *post-hoc—*analysis: DTK-test; *n* = 34; accuracy (z_*f*_ < stdTTP,*stdZ*): *p* < 0.001 sig. [corr.]]. The cumulative analysis revealed a significantly lower accuracy of z_*f*_ as compared to all other parameters [Kruskal-Wallis test; *post-hoc*—analysis: DTK-test; *n* = 66; cumulated accuracy (z_*f*_ < Tmax,stdTTP,*stdZ*): *p* < 0.001 sig. [corr.]]. A full summary is given in Table 2.

**Table 2:** Summary of quality factors (QF: median ± MAD): specificity (Spec.), sensitivity (Sens.), and accuracy (Acc.) of the Time-to-Maximum (Tmax:=T) -, the standardized Time-to-Peak (stdTTP:=S) -, the normalized TTP (z_f_) -, and the standardized and normalized TTP (stdZ:=Z)—perfusion parameter.

	**QF**	**Tmax**	**stdTTP**	**z_****f****_**	**stdZ**
Group 1 (*n* = 32; 3.0 T, TR = 1390 ms)	Spec. Sens. Acc.	0.901 ± 0.052 0.524 ± 0.187 0.874 ± 0.043	0.932 ± 0.043 0.558 ± 0.231 0.905 ± 0.038	0.713 ± 0.068^*^^T, S, Z^ 0.767 ± 0.105^*^^T, Z^ 0.721 ± 0.059^*^^T, S, Z^	0.891 ± 0.082 0.467 ± 0.218 0.861 ± 0.067
Group 2 (*n* = 34; 1.5 T, TR = 689 ms)	Spec. Sens. Acc.	0.900 ± 0.062 0.564 ± 0.192 0.870 ± 0.071	0.936 ± 0.047 0.550 ± 0.167 0.893 ± 0.048	0.722 ± 0.093^*^^S, Z^ 0.690 ± 0.164^*^^Z^ 0.718 ± 0.080^*^^S, Z^	0.926 ± 0.057 0.419 ± 0.197 0.878 ± 0.059
Cumulative (*n* = 66)	Spec. Sens. Acc.	0.901 ± 0.057 0.542 ± 0.183 0.870 ± 0.050	0.933 ± 0.046 0.558 ± 0.169 0.901 ± 0.043	0.714 ± 0.083^*^^S, Z^ 0.718 ± 0.157^*^^T, S, Z^ 0.720 ± 0.073^*^^T, S, Z^	0.917 ± 0.061 0.436 ± 0.179 0.874 ± 0.064

Except for z_f_, all perfusion parameters showed the same behavior concerning the QFs (the

* *-symbol denotes a significant difference at p < 0.001 for comparisons of z_f_ with T, S, and Z)*.

**Figure 3:**
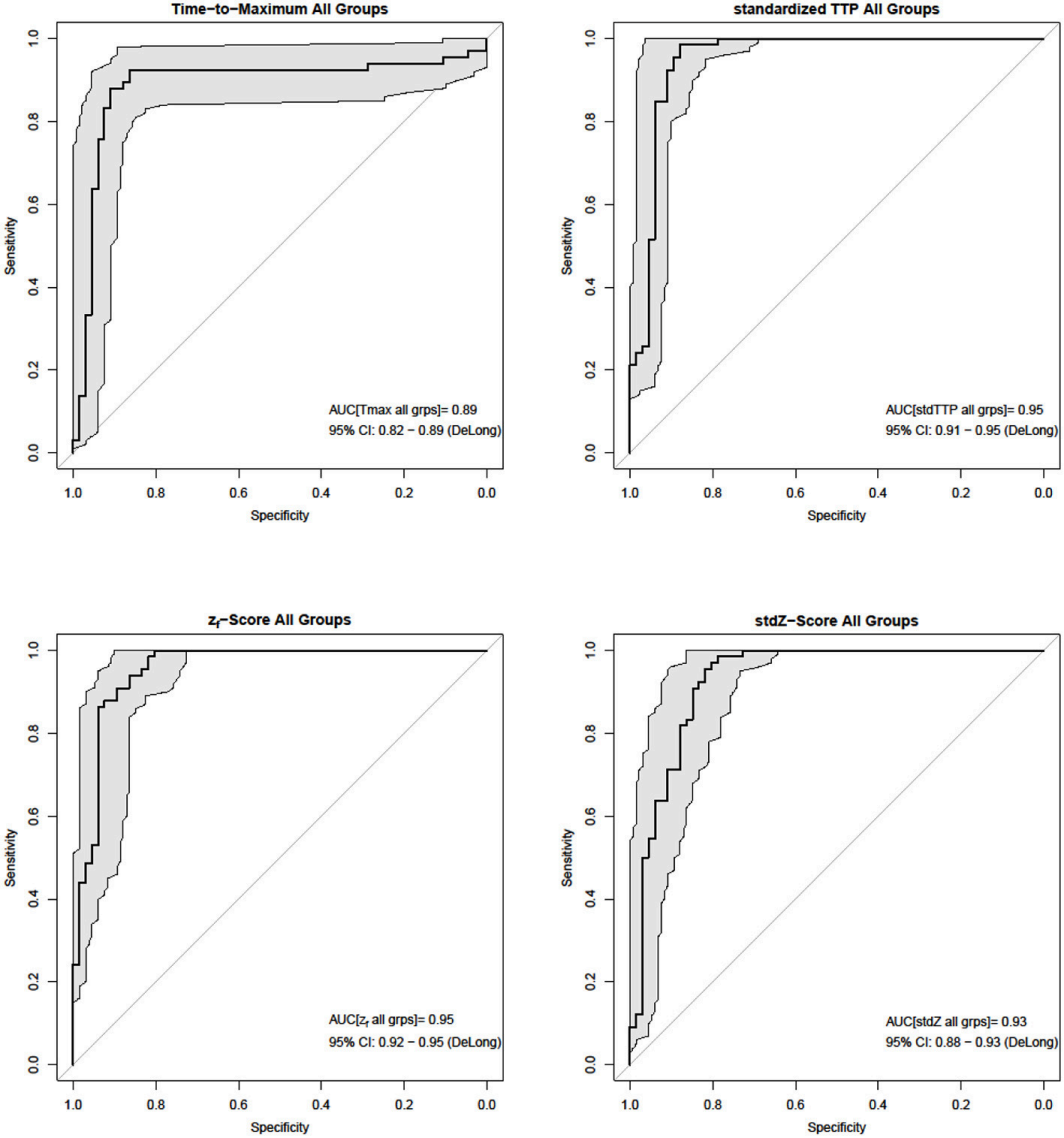
In the cumulative ROC-analysis (bold black lines and gray areas mark the 95% CI_DeLong_) of individual infarct characteristics stdTTP and *z*_*f*_ performed best (*AUC*_*stdTTP*_ = AUC_*z*_*f*__ = 0.95), while performance of stdZ (*AUC*_*stdZ*_ = 0.93) was slightly lower. Tmax (*AUC*_*Tmax*_ = 0.89) showed the lowest performance.

**Figure 4:**
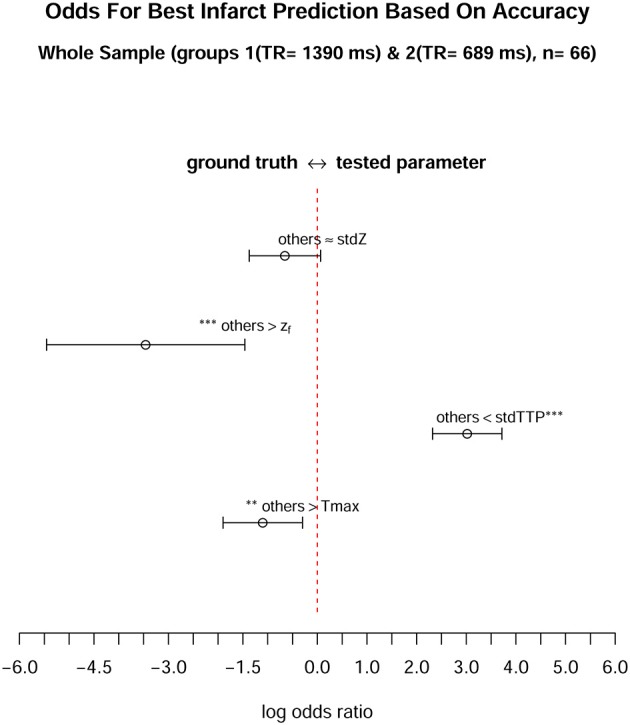
When comparing the odds of each parameter to achieve the best infarct prediction in the whole sample (*n* = 66), only the stdTTP-parameter showed a significantly high chance to correctly predict the final infarct size. The parameters were tested against a cumulative ground truth consisting of the accuracies of all other parameters (described as “others” in the figure). Accuracy was chosen as a surrogate, since it considers both, trueness and precision, respectively. All values are given on a logarithmic scale, where log odds (open mid-points) and 95%-confidence intervals (range markers) are displayed. Negative log odds **(left** side) indicate a good result in favor of the ground truth, while positive log odds (**right** side) are in favor of the tested parameter. Asterisks adjacent to a parameter or to the ground truth denote a significantly high chance to get the best infarct prediction by this quantity (^*^p < 0.05, ^**^p < 0.01, ^***^p < 0.001).

Analysis of parameter performance depending on the respective individual infarct characteristics revealed best over-all performance of z_*f*_ (ROC; z_*f*_: AUC = 0.98, CI_95%_ = 0.94–0.98) for group 1, where all other parameters also showed comparable performances (ROC; Tmax: AUC = 0.96, CI_95%_ = 0.91–0.96; stdTTP: AUC = 0.95, CI_95%_ = 0.89–0.95; *stdZ*: AUC = 0.92, CI_95%_ = 0.86–0.92). In group 2, performance of stdTTP (ROC; stdTTP: AUC = 0.95, CI_95%_ = 0.90–0.95) was highest. While z_*f*_ and *stdZ* provided comparably high results (ROC; z_*f*_: AUC = 0.93, CI_95%_ = 0.87–0.93; *stdZ*: AUC = 0.94, CI_95%_ = 0.87–0.94), performance of Tmax (ROC, Tmax: AUC = 0.82, CI_95%_ = 0.70–0.82) was lower. Cumulative assessment of perfusion parameter performance showed similar results (Figure 3). A comparably good performance of all distribution based parameters (ROC; stdTTP: AUC = 0.95, CI_95%_ = 0.91–0.95; z_*f*_: AUC = 0.95, CI_95%_ = 0.92–0.95; *stdZ*: AUC = 0.93, CI_95%_ = 0.88–0.93), but still a somewhat lower performance of Tmax (ROC, Tmax: AUC = 0.89, CI_95%_ = 0.82–0.89).The absolute frequencies in the whole sample to reach the highest accuracy in a measurement were *n* = 8 for Tmax, *n* = 48 for stdTTP, *n* = 1 for z_*f*_ and *n* = 11 for *stdZ*. Significantly high odds for the best infarct prediction in every measurement were, therefore, found only for the stdTTP-parameter, when its accuracies were compared to a cumulative ground truth consisting of the accuracies of all other parameters (odds-ratio test; stdTTP > [Tmax,z_*f*_,*stdZ*]; *p* = 2.2·10^−16^). In doing so, stdTTP showed a 6.42 times higher relative chance to obtain the best result compared to all other parameters (Figure 4). Only the *stdZ*—parameter came close to this, but failed to reach significance (odds-ratio test; *stdZ*> [Tmax,z_*f*_,stdTTP]; *p* = 0.07432).

## Discussion

In our multi-center and multi-parameter DSC-MRI study, we could not confirm superior performance of the AIF-based parameter Tmax in the assessment of ischemia compared to TDC-based parameters stdTTP, z_*f*_ and *stdZ*. Quality factors for the various perfusion parameters were not significantly different except for z_*f*_. Estimating the final infarct size stdTTP provided significantly better results than Tmax and appeared more robust to the examined variations of the DSC-sequence protocol. Moreover, all TDC-based perfusion-parameters offered unequivocal calculation- and interpretation- models, while this is not as clearly defined for Tmax. The calculation of TDC-based parameters is straight-forward, as they solely rely on the TTP-histogram directly derived from the DSC-MRI measurement. Thus, TDC-based parameters potentially display physiological alterations of brain perfusion more closely. Additionally, calculation of stdTTP, z_*f*_ and *stdZ* requires neither fitting of the actually measured TC nor the selection of an AIF since deconvolution of the TC is omitted. Given these facts, we here pose the question, whether use of less complex perfusion parameters, like the TDC-based ones, could offer more consistent and reliable clinical data about individual infarct characteristics even when acquired in different MRI-protocol settings, i.e., field strength and protocol/parameters.

All tested perfusion parameters offered an acceptable performance to differentiate ischemia from regular perfusion. As expected, higher specificity was obtained at the cost of lower sensitivity and vice versa. Powerful therapies for the treatment of acute ischemic stroke massively improved outcome for patients, thereby modifying the natural course of ischemia (Mokin et al., 2016). Consequently, perfusion measurements in acute ischemic stroke can hardly predict the exact size and shape of the final infarct, which will always be a result of the modification induced by the respective treatment combined with complex pathophysiological effects from, e.g., spontaneous recanalization, thrombus load and migration, collaterals, etc. Therefore, perfusion measurements in acute ischemia rather display ‘*individual infarct characteristics*' leading to the final infarct volume than a true infarct prediction. In this context sensitivities ranging from 0.42 to 0.77 only are comprehensible, whereas the associated specificities ranged higher between 0.72 and 0.94.

When comparing the properties of the tested perfusion parameters the behavior of z_*f*_ was most exceptional. The z_*f*_-parameter lacked specificity in both groups, but sensitivity of z_*f*_ was best. As z_*f*_ responded to the different DSC-MRI acquisition protocols comparably to the other TDC-based perfusion parameters, an effect from the DSC-MRI acquisition protocol seems unlikely. Using the IPv from the associated ***TDC***_***f***_, where the function changes its wash-out characteristics, instead of a fixed threshold to differentiate critical perfusion could explain this behavior only in part, because the same approach was used for *stdZ* that reached a significantly higher specificity and sensitivity. On the other hand, z_*f*_ results from a model-based normalization of the TTP-histogram and is, therefore, similar to absolute TTP, very susceptible to any bolus arrival delays. These delays potentially shift absolute TTP, and also z_*f*_, toward later time points in the measurement and increase, therefore, the total number of voxels marked positive for ischemia with respect to the applied threshold. In consequence, this leads to a higher chance to correctly detect a voxel of the final infarct volume, which increases sensitivity, but concurrently, as true and false positive observations increase likewise, reduces specificity (Perthen et al., 2002). For this reason accuracy, the most appropriate indicator for showing the closeness of the respective perfusion-parameter prediction to the final infarct volume, while taking true positives and negatives into account, was calculated as well. Other indicators, e.g., the Sørensen-Dice coefficient, were not considered, because by neglecting true negative voxels, severe bias due to over-weighting unspecific perfusion-parameters is introduced. Accordingly, accuracy was found lowest for z_*f*_ compared to the other perfusion parameters, where, in this aspect, the behavior of z_*f*_ seems to behave similar to absolute TTP that may also exhibit high sensitivity associated with lower specificity. Nevertheless, bolus delays contain also hemodynamic meaningful information, which may prove useful in screening cerebro-vascular disease (Zaro-Weber et al., 2010; Nasel et al., 2017).

Tmax, stdTTP, and *stdZ* exhibited sensitivities and specificities significantly higher than z_*f*_, where among the three parameters the quality factors were not significantly different. In absolute numbers, specificities for Tmax and stdTTP were similar, but sensitivity of *stdZ* was slightly lower. This might relate to the fact that, like z_*f*_, *stdZ* also employed the corresponding ***TDC***_***f***_ - IPv as critical perfusion threshold, which leads to an individual adaption of this threshold to the respective DSC-MRI measurement. Compared to a fixed threshold, it is conceivable that critical perfusion is judged more conservatively, because the TDC-model robustly eliminates global circulation effects and adapts, thereby, the threshold (Nasel et al., 2014, 2017). Fixed thresholds, on the other hand, may not sufficiently consider physiological adaptions of cerebral perfusion, as they rigidly require suitable correction of regional bolus delays and dispersion during the parameter calculation. This clearly yields a higher risk of over-, as well as, underestimation of hypoperfusion by the respective parameter.

Nevertheless, as the same threshold adaption and normalization of the TDC is inherent in both parameters, z_*f*_ and *stdZ*, the higher specificity of *stdZ* very likely results from the standardization step that, in turn, *stdZ* shares with stdTTP (Nasel et al., 2000). Surprisingly, introducing standardization of z_*f*_ immediately brings *stdZ* close to the quality performance of both, stdTTP and Tmax. This is particularly interesting as, at first glance, stdTTP and Tmax do not have much in common.

Standardization was described first for the stdTTP parameter and proved extremely robust and reliable since. StdTTP does not need selection of an AIF, as there is no deconvolution step, nor is fitting of the TC recommended. Spatiotemporal precision of stdTTP is simply controlled by TR and the acquisition matrix, both optimized for a sufficient SNR (signal-to-noise ratio) (Nasel et al., 2000, 2001, 2004). Calculation of stdTTP is straightforward, provides only one distinct solution and suits parallel computing implementations to enable real-time solutions. The slice specific time offset simulates the simultaneous arrival of the administered bolus in all slices. In regularly perfused brains this widely eliminates effects from hemodynamically less meaningful delays arising from the geometry of the vessel tree (Nasel et al., 2000). Similarly, this reduces spurious adding of extracranial bolus run time delays over the slices, e.g., in case of stenotic cerebrovascular disease (Nasel et al., 2001).

Only voxels with early (= low) absolute TTP-values at the far right side of the TDC contribute to the respective slice offset. The original rule to use just the first 3% of early enhancing voxels in each slice to obtain the offset was determined empirically. Note that this rule agrees well with the later described *z*_*f*_ = -2 score of the TDC-model. Physiologically, these voxels represent the early filling arteries in the various vascular territories (Nasel et al., 2014). From a methodical point of view, these voxels possess optimal time-concentration curves, because at this stage of the bolus distribution the dilution of the contrast medium is rather small. Thus, slice specific stdTTP-time offsets are derived from the most reliable time-contrast profiles of the DSC-measurement.

Compared to AIF-based techniques, stdTTP-time offsets consider voxels with early absolute TTP-values from all over the corresponding slice and do not revert to, more or less, distant measures from certain vascular territories or vessels. As all perfusion parameters in this study are prone to run time delays of the administered contrast bolus, a close spatiotemporal relationship between the voxel assessed and voxels contributing to the respective reference measure (AIF or offset time) used to correct for the undesirable run time delays is of utmost importance. When calculating stdTTP, the absolute TTP of voxels in a certain slice is correlated with an offset-time derived from voxels of the same slice. This establishes a close spatiotemporal relationship between offset-contributing voxels and those standardized by this offset. In this respect, stdTTP already from the beginning anticipated the necessity to set reference measures in their spatiotemporal relation reasonably close to the assessed voxel. Probably, in the calculation of Tmax replacement of sSVD with oSVD, where the latter renders Tmax more insensitive to undesired bolus delays, has a comparable effect. Yet this remains to be proven, but it is conceivable that compared to sSVD, AIF-contributing voxels selected by oSVD, inherit a closer spatio-temporally relationship to the respective voxel assessed.

The somewhat later described perfusion parameter Tmax strongly depends on modeling TC, on the selection of an AIF for deconvolution and, finally, as mentioned above, on the chosen deconvolution method (Perthen et al., 2002; Yamada, 2002; Wu et al., 2003; Christensen et al., 2009; Calamante et al., 2010; Forkert et al., 2013; Meijs et al., 2016). Both, Tmax and stdTTP, rely on a single time-based threshold for the detection of ischemia. The meanwhile frequently used 6 s threshold for Tmax was ascertained in several studies investigating acute ischemic stroke, although the discussion about the most appropriate threshold has not yet ceased (Christensen et al., 2009; Zaro-Weber et al., 2010, 2012). The 7 seconds threshold used with stdTTP was derived successively from the assessment of regular brain perfusion, stenotic carotid artery disease and acute ischemic stroke. This led to the definition of the stdTTP-triple-range model that was purely based on pathophysiological findings of cerebral perfusion. This model allows a distinct threshold interpretation that is also robust to delays introduced by stenotic cerebral disease (Nasel et al., 2000, 2001, 2004). On the other hand, as Tmax leaves much room for variations, this promotes site-specific optimization of the Tmax calculation and threshold definition. Consequently, Tmax perfusion maps based on different calculation protocols may not show identical information. This has to be considered when comparing results from groups 1 and 2, and may be one reason why Tmax did not perform better than stdTTP. Moreover, quality factors of stdTTP performed better in most cases. Accordingly, when simply asking which parameter reached the best final infarct size prediction in all measurements, based on accuracy that considers trueness and precision, only stdTTP showed a significant high chance to give the best result.

**Figure 5:**
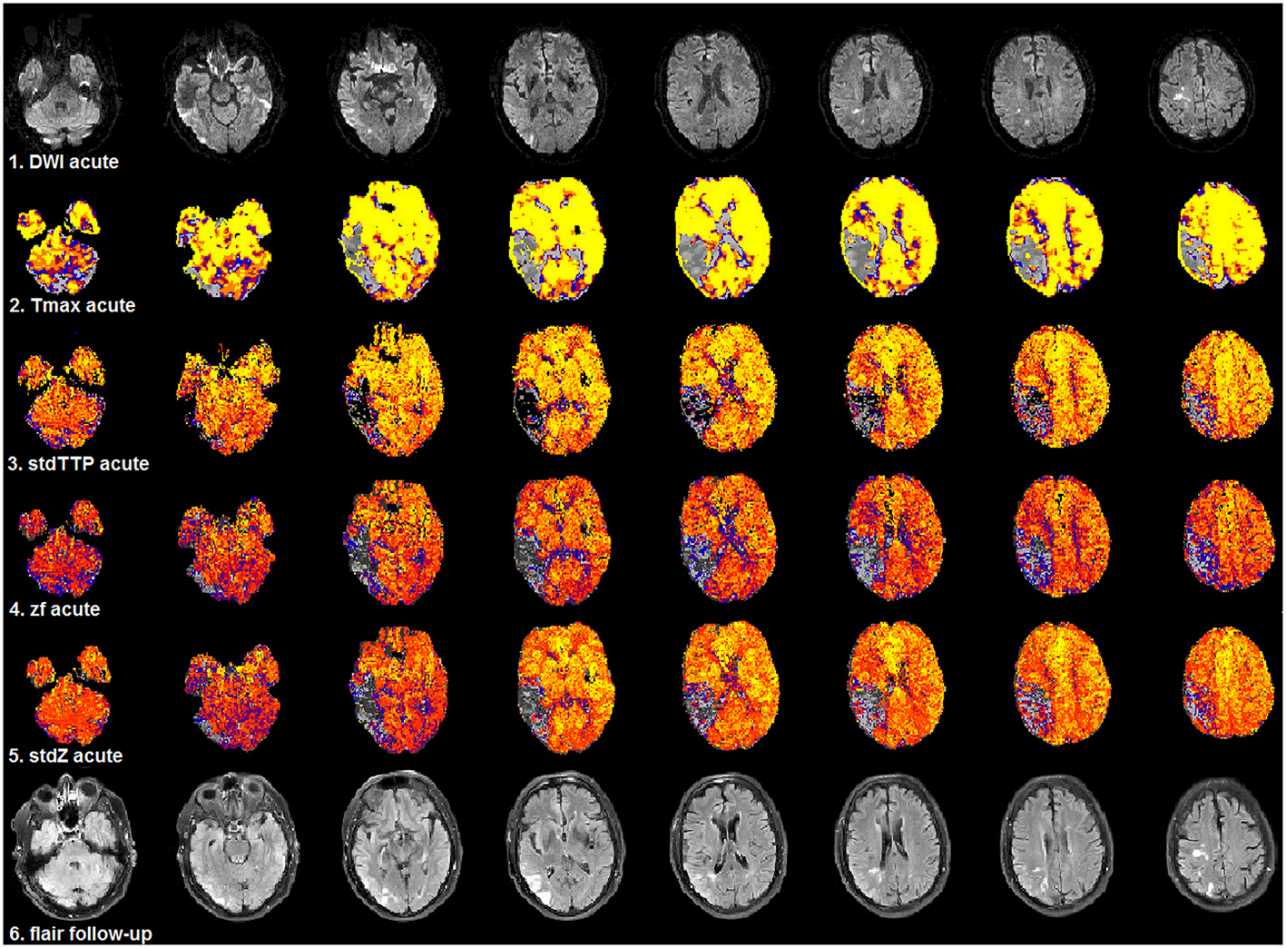
Selected patient from group 1 (3.0T, TR = 1,390 ms, stdTTP: jPerfusionModule®, Tmax: StrokeTool®) with favorable outcome after severe ischemia in the right, parieto-temporal region. Compared to acute DWI (1st row) no significant infarct progression was observed in the follow-up T2w-imaging (6th row). Initially, all perfusion parameters showed a significant mismatch using the various proposed thresholds for critical perfusion [Tmax: 6 s (2nd row); stdTTP: 7 s (3rd row); z_f_ and stdZ: IP_v_ (4th and 5th row)]. The same color look-up table (stdTTP-3 range colors; regular perfusion: yellow-red, tolerable perfusion: blue colors, critical perfusion: gray-black colors) was used for all parameter maps. The critically perfused volumes (gray-black colors) look quite similar in all maps, though, stdTTP, z_f_, and stdZ simply assess the TTP-histogram and do not need an AIF. Note that Tmax calculated with sSVD was not able to differentiate any hemodynamic aspects of the bolus distribution over time in the brain [only one color-step (yellow) is seen in regular perfused tissue]. Using stdTTP, z_f_, and stdZ this information is well-preserved (yellow to red colors showing the bolus passage over time in regular perfused tissue).

**Figure 6:**
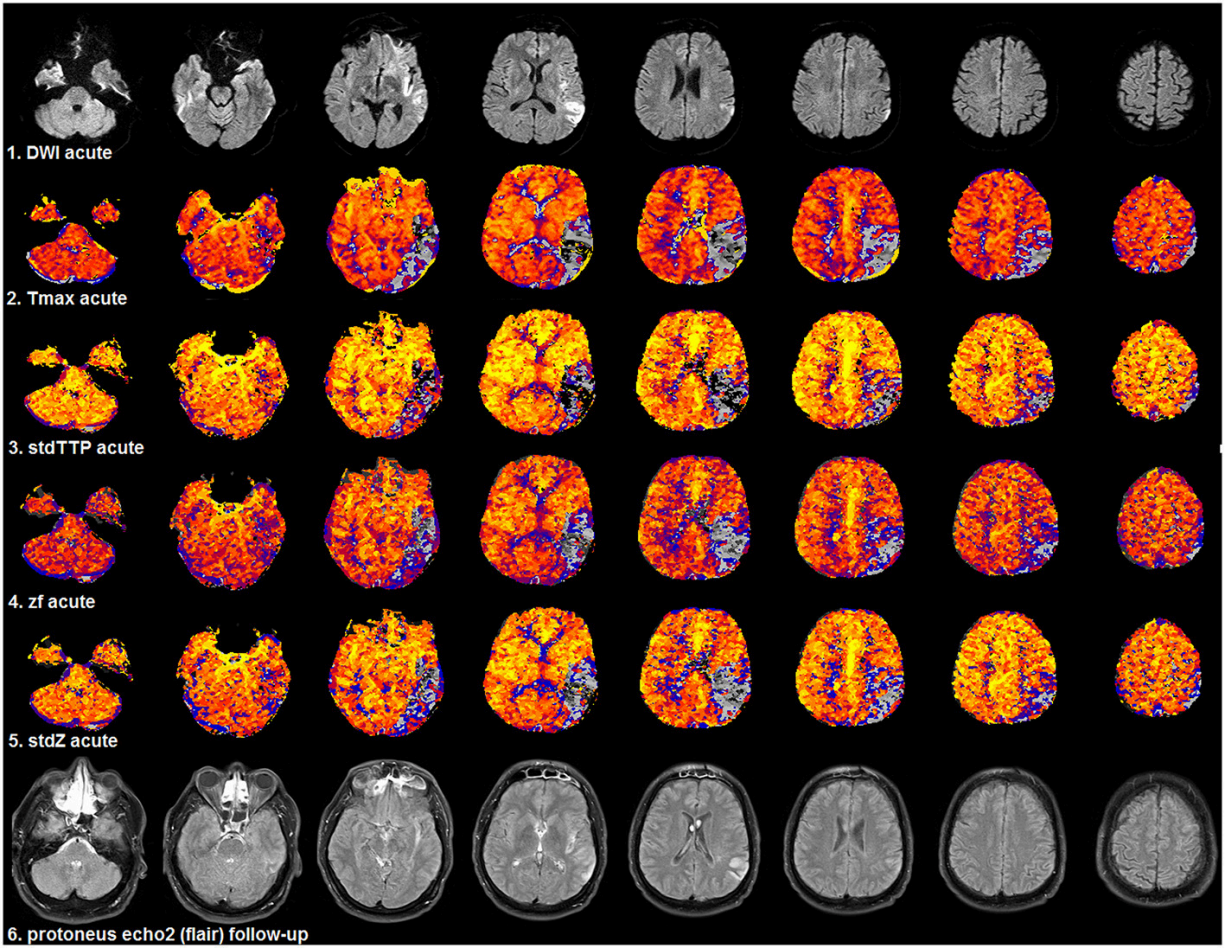
Selected patient from group 2 (1.5T, TR = 689 ms, stdTTP: jPerfusionModule®, Tmax: Olea Sphere®) suffering severe ischemia in the left, parieto-occipito-temporal region with a comparably good outcome as the patient shown in Figure 5. Compared to acute DWI (1st row) no significant infarct growth was found in the follow-up T2w-imaging (6th row). Look-up tables, meaning of colors and threshold adjustments are the same as in Figure 5. The AIF-based Tmax parameter [2nd row] did not perform better than the distribution-based parameters: stdTTP [3rd row], z_f_ [4th row], and stdZ [5th row]. The displayed critically perfused volume was comparable for all perfusion parameters (gray to black colors). Note that calculating Tmax with oSVD using the given software package preserved the hemodynamic aspects of the bolus distribution over time comparably to stdTTP, z_f_, and stdZ.

Though Tmax and stdTTP do not share much, these parameters showed a rather similar performance (Figures 5, 6). This suggests that standardization could yield an effect comparable to deconvolution. The relation between z_*f*_ and *stdZ* also supports this assumption, where standardization converged the performance of z_*f*_ to that of Tmax and stdTTP. Note that standardization alters the original TTP-distribution by correcting effects from undesired bolus run time delays basically unrelated to hemodynamic impairment. We also observed that after standardization the shape of the stdTTP-histogram was quite similar to that of Tmax, which was especially true when oSVD was used for deconvolution and the spatiotemporal resolution was sufficiently high. This effect becomes visible by the fact that the *stdZ* maps resemble very much those of stdTTP. On the other hand, the z_*f*_ distribution before standardization resembles the absolute TTP-histogram, while afterwards the *stdZ* histogram is more alike to the stdTTP or Tmax distribution (Figure 1). Further investigations are still necessary, but comparing the accuracy of z_*f*_, *stdZ*, Tmax, and stdTTP, also points into the same direction.

In absolute numbers the accuracy of Tmax, stdTTP, and *stdZ* was basically comparable in our study, while that of z_*f*_ was lower, although diagnostic performance of z_*f*_ concerning the ability to correctly detect the upcoming infarct area was better than that of Tmax as seen from the ROC analysis. This finding seems to result from the high sensitivity of z_*f*_, on the one hand, and from the great variance of specificity of Tmax, especially in group 2, on the other hand. This further supports the notion that performance of Tmax may be hampered by its inconsistent calculation protocol, leading to clearly different variances in groups 1 and 2. Interestingly, stdTTP that relies on the most simple correction method, i.e., standardization, exhibited the best and most reliable performance in both groups. The simple standardization algorithm proved most robust to influences from sequence parameters and examinations protocols, including field strength, which led to the best overall performance of stdTTP. This poses the question, whether this simple TDC-parameter would be more robust for multi-parametric approaches or a full multi-center comparison.

However, several limitations have to be noted as well, which likewise affect all parameters. Firstly, we used the infarct volume depicted in T2w-MRI at least 24 h after the event as standard of reference. Therefore, our results cannot measure absolute reliability of infarct prediction, because medical and interventional treatment following the initial perfusion scan may distinctly modify this volume. Hence, quality factors like sensitivity and specificity should be interpreted with caution. Thus, accuracy is likely more informative as it provides an estimate of closeness between the initial perfusion measurement and the correlated infarct volume depicted in follow-up examinations. Secondly, temporal alterations of the infarct volume ranging from early swelling to shrinkage in later phases are included. Therefore, we considered 'individual infarct characteristics' as a global variable incorporating innumerable pathophysiological factors like collateral capacity, thrombus migration, spontaneous recanalization, treatment delays, premedication, a.s.o., which are not fully quantifiable in their effect on the infarct volume (Kaesmacher et al., 2017). Thus, the proposed ROC analysis of the individual perfusion parameter performance based on “individual infarct characteristics” may be a reasonable way to compare the behavior of the tested parameters in the respective situation. As this allows inclusion of follow-up examinations from different time points, better estimates of the perfusion parameter practicability could be derived. In this context, we also did not perform extensive subgroup analyses with respect to the number of cases. Anyway, the study presents a direct comparison of the assessed perfusion parameters tested under exactly the same conditions. Since a perfusion parameter should lead as close as possible to the finally resulting lesion under every condition, the results appear to be a valid estimate of the practical value of Tmax, stdTTP, z_*f*_ and *stdZ*. Thirdly, we did not incorporate motion correction or other types of image quality processing into our computations in order to avoid additional bias from different correction algorithms and value interpolation. The quality of the acquired raw data was checked visually only before starting the parameter calculations, which we consider as a minor restriction. Moreover, for stdTTP, the parameter with the best performance, neither motion correction nor any other image manipulation was applied. Additionally, it has to be kept in mind that commercially available software packages used for Tmax assessment may not document each calculation step precisely, which could have had an additional effect on the results, e.g., from image smoothing, etc. In addition, we noticed a clearly different behavior of Tmax in the detection of hemodynamic aspects of the bolus distribution over time, when software from different vendors, and oSVD instead of sSVD, was used for the calculations (Figures 5, 6). When using sSVD all hemodynamic information from the step by step filling of the various vessel segments over time got lost. Theoretically, this could have a tremendous effect on the judgement of the state of the collaterals and even on comparisons of the results from different studies, when not exactly the same software with identical adjustments is used. Therefore, as far as known today no “standard software” for Tmax is available, since nearly all vendors repeatedly published new versions of their commercial packages. In contrast, for stdTTP, *z*_*f*_ and *stdZ* the software and, especially, the calculation methods remained unchanged since their first publication. Therefore, it is also not possible to claim that always the best way to calculate Tmax in the individual situation was chosen in this study, which widely results from the inconsistency of the Tmax calculation discussed above.

## Conclusion

We obtained evidence that DSC-MRI perfusion parameters stdTTP, z_*f*_, *stdZ* and directly derived from the TTP-distribution over time, without the need to select an AIF for deconvolution of TCs, show a non-inferior and more robust performance with less variation than the competitively tested AIF-based parameter Tmax. Considering the “individual infarct characteristics” as the virtual target variable of a DSC-MRI examination, TDC-based parameters performed even better than Tmax. Overall, the best results were obtained from stdTTP that yields the most simple calculation pipeline staying closest to the physiology. Since recently published large clinical trials estimate treatment success of critically ill stroke patients using the AIF-based parameter Tmax, with regard to robustness and stability also the use of distribution based parameters should be considered (Lansberg et al., 2012; Albers et al., 2018). Based on our results presented we, therefore, suggest to reanalyze available large cohort data using a wider range of methods in order to provide a more representative quantitative comparison to the stroke community.

## Ethics Statement

EK-Number: GS1-EK-4/512-2017; Study Title: Statistische Analyze zerebraler Perfusionsmessungen mittels der dynamischen Suszeptibilitätskontrast Magnetresonanzbildgebung; Investigator: Univ. Prof. C. Nasel MD Ph.D. M.Sc. (Radiologist, EDiNR), University Hospital Tulln; Specialization: Radiology, Medical Physics; Informed consent: retrospectice data analysis–informed consent was obtained, if applicable, in accordance to the ETHK; Populations: no vulnerable populations were involved in this study.

## Author Contributions

CN, UK, KV, and JF conducted the data collection. CN and EM conceptualized the study design. CN, EM, and AV contributed to the analysis and interpretation of the data. CN drafted the paper. All other authors revised it critically and approved the final version and agreed to be accountable for all aspects of this work.

### Conflict of Interest Statement

The authors declare that the research was conducted in the absence of any commercial or financial relationships that could be construed as a potential conflict of interest. The handling editor declared a shared affiliation, though no other collaboration, with one of the authors UK at time of review.

## Abbreviations

AIF: arterial input function
AUC: area under the curve
CBF: cerebral blood flow
CI_95%_: 95%-interval of confidence
DSC: dynamic susceptibility contrast
EPI: echo planar imaging
GE: gradient echo
IPv: venous inflection point
MAD: median absolute deviation
MTT: mean transit time
MRI: magnetic resonance imaging
oSVD: oscillation index regularized block-circulant SVD (→)
P-MRI: perfusion-MRI (→)
QF: quality factors
relTTP: relative TTP (→)
ROC: receiver-operator characteristics
ROI: regions of interest
rTPA: recombinant tissue plasmin activator
SVD: singular value decomposition
sSVD: standard SVD (→)
stdTTP: standardized TTP (→)
stdZ: standardized-z_f_
TC: time-concentration curve
Tmax: time-to-maximum
TTP: time-to-peak
TDC: TTP (→) distribution curve
VOI: volumes of interest.

## References

[B1] Albers G. W. Marks M. P. Kemp S. Christensen S. Tsai J. P. Ortega-Gutierrez S.. (2018). Thrombectomy for stroke at 6 to 16 hours with selection by perfusion imaging. N. Eng. J. Med. 378, 708–718. 10.1056/NEJMoa1713973 29364767 PMC6590673

[B2] Bordier C. Dojat M. Lafaye de Micheaux P. (2011). Temporal and spatial independent component analysis for fmri data sets embedded in the analyze FMRI R Package. J. Stat. Softw. 44, 1–24. 10.18637/jss.v044.i09

[B3] Boutelier T. Kudo K. Pautot F. Sasaki M. (2012). Bayesian hemodynamic parameter estimation by bolus tracking perfusion weighted imaging. IEEE Trans. Med. Imaging 31, 1381–1395. 10.1109/TMI.2012.2189890 22410325

[B4] Calamante F. Christensen S. Desmond P.M Ostergaard L. Davis S.M Connelly A. (2010). The physiological significance of the time-to-maximum (Tmax) parameter in perfusion MRI. Stroke 41, 1169–1174. 10.1161/STROKEAHA.110.580670 20413735

[B5] Carroll T. J. Teneggi V. Jobin M. Squassante L. Treyer V. Hany T. F. (2002). Absolute quantification of cerebral blood flow with magnetic resonance, reproducibility of the method, and comparison with H2O positron emission tomography. J. Cereb. Blood Flow Metab. 22, 1149–1156. 10.1097/00004647-200209000-00013 12218421

[B6] Christensen S. Mouridsen K. Wu O. Hjort N. Karstoft H. Thomalla G.. (2009). Comparison of 10 perfusion MRI parameters in 97 sub-6-hour stroke patients using voxel-based receiver operating characteristics analysis. Stroke 40, 2055–2061. 10.1161/STROKEAHA.108.546069 19359626

[B7] DeLong E. R. DeLong D. M. Clarke-Pearson D. L. (1988). Comparing the areas under two or more correlated receiver operating characteristic curves: a nonparametric approach. Biometrics 44, 837–845. 10.2307/2531595 3203132

[B8] Forkert N. D. Kaesemann P. Treszl A. Siemonsen S. Cheng B. Handels H.. (2013). Comparison of 10 TTP and Tmax estimation techniques for MR perfusion-diffusion mismatch quantification in acute stroke. Am. J. Neuroradiol. 34, 1697–1703. 10.3174/ajnr.A3460 23538410 PMC7965638

[B9] Gruen B. Leisch F. (2008). FlexMix Version 2: finite mixtures with concomitant variables and varying and constant parameters. J. Stat. Softw. 28, 1–35. 10.18637/jss.v028.i0427774042

[B10] Kaesmacher J. Maegerlein C. Kaesmacher M. Zimmer C. Poppert H. Friedrich B.. (2017). Thrombus migration in the middle cerebral artery: incidence, imaging signs, and impact on success of endovascular thrombectomy. J. Am. Heart Assoc. 6:e005149. 10.1161/JAHA.116.005149 28202431 PMC5523786

[B11] Lansberg M.G Straka M. Kemp S. Mlynash M. Wechsler L. R Jovin T. G.. (2012). MRI profile and response to endovascular reperfusion after stroke (DEFUSE 2): a prospective cohort study. Lancet Neurol. 11, 860–867. 10.1016/S.1474-4422(12)70203-X 22954705 PMC4074206

[B12] Lau M. K. (2013). DTK: Dunnett-Tukey-Kramer Pairwise Multiple Comparison Test Adjusted for Unequal Variances and Unequal Sample Sizes. R package version 3.5. Available online at: http://CRAN.R-project.org/package=DTK; accessed: 2015.07.22

[B13] Meijs M. Christensen S. Lansberg M. G. Albers G. W. Calamante F (2016). Analysis of perfusion MRI in stroke: To deconvolve, or not to deconvolve. Magn. Reson. Med. 76, 1282–1290. 10.1002/mrm.26024 26519871

[B14] Meyer D. Zeileis A. Hornik K. (2017). vcd: Visualizing Categorical Data. R package, version: 1.4-4. Available online at: https://cran.r-project.org/web/packages/vcd/index.html (Accessed: September 29, 2018).

[B15] Mokin M. Rojas H. Levy E. I. (2016). Randomized trials of endovascular therapy for stroke — impact on stroke care. Nat. Rev. Neurol. 12, 86–94. 10.1038/nrneurol.2015.240 26782336

[B16] Nasel C. (2005). Protoneus-sequence: extended fluid-attenuated inversion recovery MR imaging without and with contrast enhancement. Eur. J. Radiol. 55, 219–223. 10.1016/j.ejrad.2004.11.008 16036150

[B17] Nasel C. Azizi A. Veintimilla A. Mallek R. Schindler E. (2000). A standardized method of generating time-to-peak perfusion maps in dynamic-susceptibility contrast-enhanced MR imaging. AJNR Am. J. Neuroradiol. 21, 1195–1198. 10954268 PMC8174923

[B18] Nasel C. Azizi A. Wilfort A. Mallek R. Schindler E. (2001). Measurement of time-to-peak parameter by use of a new standardization method in patients with stenotic or occlusive disease of the carotid artery. AJNR Am. J. Neuroradiol. 22, 1056–1061. 11415897 PMC7974788

[B19] Nasel C. Boubela R. Kalcher K. Moser E. (2017). Normalised time-to-peak-distribution curves correlate with cerebral white matter hyperintensities - could this improve early diagnosis? J. Cereb. Blood Flow Metab. 37, 444–455. 10.1177/0271678X16629485 26823469 PMC5256485

[B20] Nasel C. Kalcher K. Boubela R. Moser E. (2014). Improved quantification of cerebral hemodynamics using individualized time thresholds for assessment of peak enhancement parameters derived from dynamic susceptibility contrast enhanced magnetic resonance imaging. PLoS ONE 9:e114999. 10.1371/journal.pone.0114999 25521121 PMC4270773

[B21] Nasel C. Kronsteiner N. Schindler E. Kreuzer S. Gentzsch S. (2004). Standardized time to peak in ischemic and regular cerebral tissue measured with perfusion MR imaging. AJNR Am. J. Neuroradiol. 25, 945–950. 15205128 PMC7975663

[B22] Ogle D. (2018). FSA: Simple Fisheries Stock Assessment Methods. R package, version: 0.8.22. Available online at: https://cran.r-project.org/web/packages/FSA/index.html (Accessed: November 22, 2018).

[B23] Olivot J.M Mlynash M. Thijs V.N Purushotham A. Kemp S. Lansberg M. G.. (2009). Geography, structure, and evolution of diffusion and perfusion lesions in diffusion and perfusion imaging evaluation for understanding stroke evolution (DEFUSE). Stroke 40, 3245–3251. 10.1161/STROKEAHA.109.558635 19679845 PMC2753724

[B24] Ostergaard L. (2005). Principles of cerebral perfusion imaging by bolus tracking. J. Magn. Reson. Imaging 22, 710–717. 10.1002/jmri.20460 16261573

[B25] Ostergaard L. Weisskoff R. M. Chesler D. A. Gyldensted C. Rosen B. (1996). High resolution measurement of cerebral blood flow using intravascular tracer bolus passages. Part I: mathematical approach and statistical analysis. Magn. Reson. Med. 36, 715–725. 10.1002/mrm.1910360510 8916022

[B26] Perthen J. E. Calamante F. Gadian D. G. Connelly A. (2002). Is quantification of bolus tracking MRI reliable without deconvolution? Magn. Reson. Med. 47, 61–67. 10.1002/mrm.10020 11754443

[B27] R Development CoreTeam (2015). R: A Language and Environment for Statistical Computing. Vienna: R Foundation for Statistical Computing.

[B28] Robin X. Turck N. Hainard A. Tiberti N. Lisacek F. Sanchez J. C.. (2011). pROC: an open-source package for R and S+ to analyze and compare ROC curves. BMC Bioinformatics 12:77. 10.1186/1471-2105-12-77 21414208 PMC3068975

[B29] Rorden C. Brett M. (2000). Stereotaxic display of brain lesions. Behav. Neurol. 12, 191–200. 10.1155/2000/421719 11568431

[B30] World Medical Association (2014). Declaration of Helsinki. WMA: World Medical Association.

[B31] Wu O. Østergaard L. Weisskoff R. M. Benner T. Rosen B. R. Sorensen A. (2003). Tracer arrival timing-insensitive technique for estimating flow in MR perfusion-weighted imaging using singular value decomposition with a block-circulant deconvolution matrix. Magn. Reson. Med. 50, 164–174. 10.1002/mrm.10522 12815691

[B32] Yamada K. (2002). Magnetic resonance perfusion-weighted imaging of acute cerebral infarction: effect of the calculation methods and underlying vasculopathy. Stroke 33, 87–94. 10.1161/hs0102.101893 11779894

[B33] Zaro-Weber O. Moeller-Hartmann W. Heiss W. D. Sobesky J. (2009). The performance of MRI-based cerebral blood flow measurements in acute and subacute stroke compared with 15O-water positron emission tomography: identification of penumbral flow. Stroke 40, 2413–2421. 10.1161/STROKEAHA.108.540914 19461037

[B34] Zaro-Weber O. Moeller-Hartmann W. Heiss W. D. Sobesky J. (2010). Maps of time to maximum and time to peak for mismatch definition in clinical stroke studies validated with positron emission tomography. Stroke 41, 2817–2821. 10.1161/STROKEAHA.110.594432 21030699

[B35] Zaro-Weber O. Moeller-Hartmann W. Heiss W. D. Sobesky J. (2012). Influence of the arterial input function on absolute and relative perfusion-weighted imaging penumbral flow detection: a validation with O-water positron emission tomography. Stroke 43, 378–385. 10.1161/STROKEAHA.111.635458 22135071

